# Personal Cancer Genome Reporter: variant interpretation report for precision oncology

**DOI:** 10.1093/bioinformatics/btx817

**Published:** 2017-12-20

**Authors:** Sigve Nakken, Ghislain Fournous, Daniel Vodák, Lars Birger Aasheim, Ola Myklebost, Eivind Hovig

**Affiliations:** 1 Norwegian Cancer Genomics Consortium, Department of Tumor Biology, Institute for Cancer Research, Oslo University Hospital, Norway; 2 Bioinformatics Core Facility, Department of Core Facilities, Institute for Cancer Research, Oslo University Hospital, Norway; 3 Department of Clinical Science, University of Bergen, Norway; 4 Department of Informatics, University of Oslo, Norway; 5 Institute for Cancer Genetics and Informatics, Oslo University Hospital, Norway

## Abstract

**Summary:**

Individual tumor genomes pose a major challenge for clinical interpretation due to their unique sets of acquired mutations. There is a general scarcity of tools that can (i) systematically interrogate cancer genomes in the context of diagnostic, prognostic, and therapeutic biomarkers, (ii) prioritize and highlight the most important findings and (iii) present the results in a format accessible to clinical experts. We have developed a stand-alone, open-source software package for somatic variant annotation that integrates a comprehensive set of knowledge resources related to tumor biology and therapeutic biomarkers, both at the gene and variant level. Our application generates a tiered report that will aid the interpretation of individual cancer genomes in a clinical setting.

**Availability and implementation:**

The software is implemented in Python/R, and is freely available through Docker technology. Documentation, example reports, and installation instructions are accessible via the project GitHub page: https://github.com/sigven/pcgr.

**Supplementary information:**

Supplementary data are available at *Bioinformatics* online.

## 1 Introduction

A range of different tools have been developed for functional annotation of genomic variants (McLaren *et al.*, 2016; Ramos *et al.*, 2015; Wang *et al.*, 2010). Although some have a specific focus on the oncology domain (e.g. *Oncotator*), they are all largely targeted towards the research community. Existing solutions offer limited support for summaries and reports at the level of individual cancer genomes, particularly when it comes to clinical relevance. Moreover, the degree of quality control and update frequency of annotation resources vary considerably. Recently, we have seen significant development of databases that harvest reports from the scientific literature about cancer genome variants and their particular relationships to tumorigenesis, druggability and clinical outcomes. These include the Database of Curated Mutations (DoCM), The Drug Gene Interaction Database (DGIdb) and most importantly the community-driven database of Clinical Interpretations of Variants in Cancer (CIViC) (Ainscough *et al.*, 2016; Griffith *et al.*, 2017; Wagner *et al.*, 2016). Other resources covering known cancer mutation hotspots, mutational signatures and predicted driver mutations have also emerged, which collectively indicate potential underlying mechanisms of tumor development and relevance for different treatment regimes (Chang *et al.*, 2016; Gonzalez-Perez *et al.*, 2013; Secrier *et al.*, 2016).

We have developed the Personal Cancer Genome Reporter (PCGR), a software package for the generation of clinically interpretable reports of individual cancer genomes. The software extends basic variant annotations from Variant Effect Predictor (VEP) with oncology-relevant annotations retrieved flexibly with *vcfanno* (Pedersen *et al.*, 2016), and produces interactive HTML reports that are intended for clinical translation.

## 2 PCGR workflow

The pipeline for generation of personal cancer genome reports comprises four major steps: (i) basic variant consequence annotation using VEP, (ii) allele-specific annotation for precision oncology using *vcfanno*, (iii) functional and cancer-focused gene annotation and (iv) prediction of MSI status, estimation of mutational signature contributions, summary, prioritization and reporting with the R language and R markdown templates (Supplementary Fig. S1). All software components are integrated and provided by means of the Docker technology, implying that all underlying dependencies are packaged into a standardized software container. The Docker solution was chosen to offer individual labs a simple installation of PCGR in their in-house workflow for high-throughput analysis of tumor genomes. The application comes with an annotation data bundle, which we plan to update on a quarterly basis. The human genome assembly GRCh37 is currently supported.

The PCGR workflow accepts two types of input files: (i) a single-sample VCF file encoding the genomic coordinates of somatic SNVs/Indels and (ii) a basic somatic copy number segment file that encodes chromosomal segment locations and their log-2 ratios (specific requirements are given in the GitHub documentation).

For SNVs and Indels encoded in a VCF file, VEP is utilized to determine variant consequence information. For the sake of simplicity and ease of use, the VEP annotation is run with a fixed set of parameters that includes all gene cross-references, protein domain annotations, and overlap with regulatory regions, using GENCODE as the underlying gene transcript model. All transcript-dependent consequences per variant are retained in the VEP-annotated VCF file, and the consequence block of highest functional relevance (as provided by VEP’s *–flag_pick* option) is flagged for further downstream analysis. Next, *vcfanno* is applied on multiple variant databases in parallel in order to enrich each somatic call with allele-specific annotations that are directly or indirectly relevant for clinical interpretation. These allele-specific annotations include, (i) pathogenicity predictions for splice-site and missense variants by multiple algorithms, (ii) overlap with known mutational cancer hotspots and previously predicted driver mutations, (iii) tissue/tumor type frequency in case of previously detected somatic variants, (iv) known disease/cancer associations and (v) clinical evidence items of relevance for diagnosis, prognosis, predisposition, or drug sensitivity/resistance.

In the third step of the workflow, gene-level annotations are aggregated. Known proto-oncogenes and tumor suppressors are marked, as are other genes implicated (either by prediction or curation) in cancer (Piñero *et al.*, 2017). Antineoplastic agents and their molecular targets are also annotated. Each gene is finally assigned a score that reflects its relative strength of association to cancer in the biomedical literature, enabling ranking of novel variants according to functional relevance (Rocco *et al.*, 2017). The annotation of copy number aberrations is limited to this third step, in which gene transcripts that intersect gained or depleted segments are identified, and clinical and etiologic cancer associations are retrieved.

The final and fourth step of the PCGR workflow summarises and prioritizes the annotated variants in a structured and interactive report, adopting recently proposed recommendations (Dienstmann *et al.*, 2014; Ritter *et al.*, 2016). Specifically, a tiered report is constructed, starting from actionable markers in *Tier 1*, toward aberrations relevant for tumorigenesis in *Tier 2 and 3*, and ending with variants of unknown functional relevance in *Tier 4 and 5*. In addition to the tier structure, mutated genes in *Tier 3-5* are prioritized by means of the above-mentioned literature-derived score for oncogenic potential, which draws attention to the most relevant findings. Finally, the report offers optional prediction of microsatellite instability, in addition to estimates of known mutational signatures present in the tumor, and their associated underlying etiologies (Rosenthal *et al.*, 2016). An example report for a breast cancer genome is found within the Supplementary Material.

## 3 Discussion

We have utilized the Docker technology to develop a report engine for clinical interpretation of cancer genomes. The tool has a particular focus on actionable, coding variants, i.e. variants found through exome sequencing. The Cancer Genome Interpreter (CGI, https://cancergenomeinterpreter.org) can identify tumor alterations that are therapeutically actionable, similar to the functionality that is implemented in PCGR. CGI is a web-based solution, while PCGR is a stand-alone annotation engine intended for integration in tumor sequencing pipelines. Moreover, PCGR exploits the total spectrum of tumor variants to compute additional measures that can inform precision therapy, i.e. MSI status, mutational signatures, and mutational burden.

Matching biomarker results more stringently to the cancer type of the query will be prioritized in future extensions of PCGR. Furthermore, we foresee that the report can be significantly strengthened through the addition of other molecular profiling datasets, such as gene expression. Expression will not only add an important layer on top of results found at the DNA level, it can also add significant value towards assessment of therapeutic potential by other analyses, such as inference of cell type composition in the tumor tissue, and *in silico* predictions of drug response.

## Supplementary Material

Supplementary Data

## References

[btx817-B1] Ainscough B.J. et al (2016) DoCM: a database of curated mutations in cancer. Nat. Methods, 13, 806–807. 27684579 10.1038/nmeth.4000 PMC5317181

[btx817-B2] Chang M.T. et al (2016) Identifying recurrent mutations in cancer reveals widespread lineage diversity and mutational specificity. Nat. Biotechnol., 34, 155–163. 26619011 10.1038/nbt.3391 PMC4744099

[btx817-B3] Dienstmann R. et al (2014) Standardized decision support in next generation sequencing reports of somatic cancer variants. Mol. Oncol., 8, 859–873. 24768039 10.1016/j.molonc.2014.03.021 PMC5528527

[btx817-B4] Gonzalez-Perez A. et al (2013) IntOGen-mutations identifies cancer drivers across tumor types. Nat. Methods, 10, 1081–1082. 24037244 10.1038/nmeth.2642 PMC5758042

[btx817-B5] Griffith M. et al (2017) CIViC is a community knowledgebase for expert crowdsourcing the clinical interpretation of variants in cancer. Nat. Genet., 49, 170–174. 28138153 10.1038/ng.3774 PMC5367263

[btx817-B6] McLaren W. et al (2016) The ensembl variant effect predictor. Genome Biol., 17, 122. 27268795 10.1186/s13059-016-0974-4 PMC4893825

[btx817-B7] Pedersen B.S. et al (2016) Vcfanno: fast, flexible annotation of genetic variants. Genome Biol., 17, 118. 27250555 10.1186/s13059-016-0973-5 PMC4888505

[btx817-B8] Piñero J. et al (2017) DisGeNET: a comprehensive platform integrating information on human disease-associated genes and variants. Nucleic Acids Res., 45, D833–D839. 27924018 10.1093/nar/gkw943 PMC5210640

[btx817-B9] Ramos A.H. et al (2015) Oncotator: cancer variant annotation tool. Hum. Mutat., 36, E2423–E2429. 25703262 10.1002/humu.22771 PMC7350419

[btx817-B10] Ritter D.I. et al (2016) Somatic cancer variant curation and harmonization through consensus minimum variant level data. Genome Med., 8, 117. 27814769 10.1186/s13073-016-0367-z PMC5095986

[btx817-B11] Rocco P. et al (2017) OncoScore: a novel, Internet-based tool to assess the oncogenic potential of genes. Sci. Rep., 7, 46290. 28387367 10.1038/srep46290 PMC5384236

[btx817-B12] Rosenthal R. et al (2016) deconstructSigs: delineating mutational processes in single tumors distinguishes DNA repair deficiencies and patterns of carcinoma evolution. Genome Biol., 17, 31. 26899170 10.1186/s13059-016-0893-4 PMC4762164

[btx817-B13] Secrier M. et al (2016) Mutational signatures in esophageal adenocarcinoma define etiologically distinct subgroups with therapeutic relevance. Nat. Genet., 48, 1131–1141. 27595477 10.1038/ng.3659 PMC5957269

[btx817-B14] Wagner A.H. et al (2016) DGIdb 2.0: mining clinically relevant drug-gene interactions. Nucleic Acids Res., 44, D1036–D1044. 26531824 10.1093/nar/gkv1165 PMC4702839

[btx817-B15] Wang K. et al (2010) ANNOVAR: functional annotation of genetic variants from high-throughput sequencing data. Nucleic Acids Res., 38, e164. 20601685 10.1093/nar/gkq603 PMC2938201

