# Supplementary material for: Personal Cancer Genome Reporter: variant interpretation report for precision oncology
Source: Bioinformatics. 2017 Dec 20;34(10):1778–80. doi: 10.1093/bioinformatics/btx817 (PMC5946881; doi:10.1093/bioinformatics/btx817)
Supplement: Supplementary Data [file btx817_supp.zip › btx817-suppl_data/tumor_sample.BRCA.0.5.0.pcgr.html]

Cancer genome sequencing report for precision oncology - tumor\_sample.BRCA


# Cancer genome sequencing report for precision oncology - tumor\_sample.BRCA

#### *November 14, 2017*

## Annotation sources

The annotated variants are based on the following underlying tools and knowledge resources:

- VEP v90 - Variant Effect Predictor release 90 (GENCODE v27 as the gene reference dataset)
- dbNSFP - database of non-synonymous functional predictions (v3.4 (March 2017))
- TCGA - The Cancer Genome Atlas (release 9.0 (October 24th 2017))
- gnomAD - germline variant frequencies exome-wide (March 2017)
- dbSNP - database of short genetic variants (build 147 (April 2016))
- 1000Genomes - germline variant (phase 3 (20130502))
- ClinVar - database of clinically related variants (November 2017 (20171102))
- DoCM - database of curated mutations (v3.2 (April 2016))
- CIViC - clinical interpretations of variants in cancer (November 11th 2017)
- CBMDB - Cancer Biomarkers database (November 11th 2017)
- IntOGen catalog of driver mutations - (2016.05)
- DisGeNET - Database of gene-disease associations (v5.0 (May 2017))
- Cancer Hotspots - a resource for statistically significant mutations in cancer (2016)
- UniProt/SwissProt KnowledgeBase - resource on protein sequence and functional information (release 2017\_10)
- Pfam - database of protein families and domains (v31.0 (March 2017))
- DGIdb - database of targeted antineoplastic drugs (v3.0 (September 2017))
- TSGene - tumor suppressor/oncogene database (v2.0 (2016))
- KEGG pathways - KEGG pathway database (September 29th 2017)
- *PCGR software version*: 0.5.0

## Somatic SNVs/InDels

Input mode: tumor vs. control

### Summary statistics

- Number of SNVs: **195**
- Number of InDels: **0**
- Number of coding variants: **126**
- Number of noncoding variants: **69**

### Tumor mutational burden (TMB)

The size of the targeted genomic region has been defined as: **40 Mb**. For estimation of TMB, we used same approach as was outlined in a recent study (Chalmers et al. 2017), i.e. counting all coding, somatic base substitutions and indels in the targeted regions, including synonymous alterations.

Estimated mutational burden: **4.1** **mutations/Mb**

### Tier statistics

The prioritization of SNV/InDels in the report is done according to a five-tiered structure, similar to proposed recommendations (Dienstmann et al. 2014), also adopting the MLVD framework for description of clinically relevant cancer variants (Ritter et al. 2016).

- Tier 1 - actionable variants: **1**
- Tier 2 - cancer hotspots, disease-causing, or predicted drivers: **3**
- Tier 3 - cancer gene variants: **9**
- Tier 4 - other coding variants: **113**
- Tier 5 - non-coding variantss: **69**

### Global distribution - allelic support

### Global variant browser

The table below permits filtering of the total SNV/InDel set by various criteria.

**NOTE 1**: The filtering applies to this table only, and not to the tier-specific tables below.

**NOTE 2**: Filtering on sequencing depth/allelic fraction depends on input specified by user (VCF INFO tags).

Tier

Sequencing depth tumor

Allelic fraction tumor

Consequence

Call confidence

### Tier 1 - Genomic biomarkers for diagnosis, prognosis, predisposition, and drug response

- A total of **1** unique, somatic variant(s) in the tumor sample can be mapped to genomic biomarkers in the database for clinical interpretations of variants in cancer, CIViC or Cancer bioMarkers database, with the following number of evidence items:
  - Tier 1 - Predictive: **20** evidence items linked to drug sensitivity/resistance
  - Tier 1 - Prognostic: **1** evidence items linked to prognosis
  - Tier 1 - Diagnostic: **0** evidence items linked to diagnosis
  - Tier 1 - Predisposing: **0** evidence items linked to predisposition

#### Predictive biomarkers

Cancer type

Clinical significance

Evidence level

Biomarker mapping

Therapeutic context

The table below lists all variant-evidence item associations:

#### Prognostic biomarkers

Cancer type

Clinical significance

Evidence level

Biomarker mapping

The table below lists all variant-evidence item associations:

#### Diagnostic biomarkers

*No variant-evidence item associations found.*

#### Predisposition biomarkers

*No variant-evidence item associations found.*

### Tier 2 - Other cancer mutation hotspots, curated mutations, or predicted driver mutations

- A total of **3** unique, somatic variant(s) in the tumor sample are curated as disease-causing, predicted as driver mutations, or occur in known cancer mutation hotspots.

#### Known mutation hotspots

The table below lists variants detected as mutation hotspots according to cancerhotspots.org:

#### Other disease-causing mutations

*No variants found.*

#### Other predicted driver mutations

The table below lists all other variants in Tier 2 predicted as cancer driver mutations in Intogen’s catalog of driver mutations:

### Tier 3 - Other coding mutations in proto-oncogenes or tumor suppressor genes

- A total of **9** unique, somatic variant(s) in the tumor sample are found within known proto-oncogenes or tumor suppressor genes.

### Tier 4 - Other coding mutations

- A total of **113** unique, coding somatic variant(s) are also found in the tumor sample.

### Tier 5 - Non-coding mutations

- A total of **69** unique, somatic variant(s) are also found in the tumor sample.

## Somatic CNA analysis

### Segments - amplifications and homozygous deletions

The following user-defined thresholds determine copy number aberrations shown here:

- **Copy number amplifications**: Log(2) ratio >= 0.8
- **Homozygous deletions**: Log(2) ratio <= -0.8

A total of **133** unfiltered aberration segments satisfied the above criteria.

A total of  copy number segments satisfy the current filtering criteria.

Log-ratio

Event type

focal

Cytoband

### Proto-oncogenes subject to copy number amplifications

A total of **4** proto-oncogenes are completely covered (i.e. transcript overlapping 100%) by genomic segments subject to amplifications.

### Tumor suppressor genes subject to homozygous deletions

A total of **0** tumor suppressor genes are completely covered (i.e. transcript overlapping 100%) by genomic segments subject to homozygous deletions.

### Copy number aberrations as biomarkers for prognosis, diagnosis, predisposition, and drug response

A total of **1** aberrations are associated with clinical evidence items in the database for clinical interpretations of variants in cancer, CIViC, with the following number of evidence items:

- Predictive: **51** evidence items linked to drug sensitivity/resistance
- Prognostic: **1** evidence items
- Diagnostic: **0** evidence items
- Predisposing: **0** evidence items

#### Predictive biomarkers

Cancer type

Clinical significance

Evidence level

Therapeutic context

Log-ratio

The table below lists all variant-evidence item associations:

#### Prognostic biomarkers

Cancer type

Clinical significance

Evidence level

Log-ratio

The table below lists all variant-evidence item associations:

#### Diagnostic biomarkers

*No variant-evidence item associations found.*

#### Predisposition biomarkers

*No variant-evidence item associations found.*

## MSI status

Microsatellite instability (MSI) is the result of impaired DNA mismatch repair and constitutes a cellular phenotype of clinical significance in many cancer types, most prominently colorectal cancers, stomach cancers, endometrial cancers, and ovarian cancers (Cortes-Ciriano et al., 2017). We have built a statistical MSI classifier from somatic mutation profiles that separates *MSI.H* (MSI-high) from *MSS* (MS stable) tumors. The MSI classifier was trained using 1000 exome-sequenced TCGA tumor samples with known MSI status (i.e. assayed from mononucleotide markers), and achieved 90.2% sensitivity and 99.7% specificity on an independent test set of 427 samples. Details of the MSI classification approach can be found here.

- *Predicted MSI status for tumor\_sample.BRCA*: **MSS (Microsatellite stable)**

### Supporting evidence I: indel fraction among somatic calls

The plot below illustrates the fraction of indels among all calls in *tumor\_sample.BRCA* (black dashed line) along with the distribution of indel fractions for TCGA samples (colorectal, endometrial, ovarian, stomach) with known MSI status assayed from mononucleotide markers ( *MSI.H* = high microsatellite instability, *MSS* = microsatellite stable):

### Supporting evidence II: coding mutations in MSI-associated genes

*No variants found.*

## Mutational signatures

The set of somatic mutations observed in a tumor reflects the varied mutational processes that have been active during its life history, providing insights into the routes taken to carcinogenesis. Exogenous mutagens, such as tobacco smoke and ultraviolet light, and endogenous processes, such as APOBEC enzymatic family functional activity or DNA mismatch repair deficiency, result in characteristic patterns of mutation (i.e. distinct patterns of substitution types in specific seqence contexts). Importantly, recent studies show that mutational signatures could have significant clinical impact in certain tumor types (Dong et al., 2016, Secrier et al., 2016, Kim et al., 2016)

Here, we apply the deconstructSigs package (Rosenthal et al., 2013) to delineate the known mutational signatures in a single tumor. This package compares the patterns of mutations observed in a single tumor with a large set of estimated signatures found across tumor types (Alexandrov et al., 2013, Alexandrov et al., 2013).

A total of **n = 195** SNVs were used for the mutational signature analysis of this tumor.

Given an input tumor profile and reference input signatures (i.e. 30 mutational signatures detected by Sanger/COSMIC), deconstructSigs iteratively infers the weighted contributions of each reference signature until an empirically chosen error threshold is reached. In the plots below, the *top panel* is the tumor mutational profile displaying the fraction of mutations found in each trinucleotide context, the *middle panel* is the reconstructed mutational profile created by multiplying the calculated weights by the signatures, and the *bottom panel* is the error between the tumor mutational profile and reconstructed mutational profile. The piechart shows the relative contribution of each signature in the sample.

### Detected mutational signatures - proposed underlying aetiologies

## References

Alexandrov, Ludmil B, Serena Nik-Zainal, David C Wedge, Samuel A J R Aparicio, Sam Behjati, Andrew V Biankin, Graham R Bignell, et al. 2013. “Signatures of Mutational Processes in Human Cancer.” *Nature* 500 (7463): 415–21.

Alexandrov, Ludmil B, Serena Nik-Zainal, David C Wedge, Peter J Campbell, and Michael R Stratton. 2013. “Deciphering Signatures of Mutational Processes Operative in Human Cancer.” *Cell Rep.* 3 (1): 246–59.

Chalmers, Zachary R, Caitlin F Connelly, David Fabrizio, Laurie Gay, Siraj M Ali, Riley Ennis, Alexa Schrock, et al. 2017. “Analysis of 100,000 Human Cancer Genomes Reveals the Landscape of Tumor Mutational Burden.” *Genome Med.* 9 (1): 34.

Cortes-Ciriano, Isidro, Sejoon Lee, Woong-Yang Park, Tae-Min Kim, and Peter J Park. 2017. “A Molecular Portrait of Microsatellite Instability Across Multiple Cancers.” *Nat. Commun.* 8 (6~jun): 15180.

Dienstmann, Rodrigo, Fei Dong, Darrell Borger, Dora Dias-Santagata, Leif W Ellisen, Long P Le, and A John Iafrate. 2014. “Standardized Decision Support in Next Generation Sequencing Reports of Somatic Cancer Variants.” *Mol. Oncol.* 8 (5): 859–73.

Dong, Fei, Phani K Davineni, Brooke E Howitt, and Andrew H Beck. 2016. “A BRCA1/2 Mutational Signature and Survival in Ovarian High-Grade Serous Carcinoma.” *Cancer Epidemiol. Biomarkers Prev.* 25 (11): 1511–6.

Kim, Jaegil, Kent W Mouw, Paz Polak, Lior Z Braunstein, Atanas Kamburov, Grace Tiao, David J Kwiatkowski, et al. 2016. “Somatic ERCC2 Mutations Are Associated with a Distinct Genomic Signature in Urothelial Tumors.” *Nat. Genet.* 48 (6): 600–606.

Ritter, Deborah I, Sameek Roychowdhury, Angshumoy Roy, Shruti Rao, Melissa J Landrum, Dmitriy Sonkin, Mamatha Shekar, et al. 2016. “Somatic Cancer Variant Curation and Harmonization Through Consensus Minimum Variant Level Data.” *Genome Med.* 8 (1): 117.

Rosenthal, Rachel, Nicholas McGranahan, Javier Herrero, Barry S Taylor, and Charles Swanton. 2016. “DeconstructSigs: Delineating Mutational Processes in Single Tumors Distinguishes DNA Repair Deficiencies and Patterns of Carcinoma Evolution.” *Genome Biol.* 17 (1): 31.

Secrier, Maria, Xiaodun Li, Nadeera de Silva, Matthew D Eldridge, Gianmarco Contino, Jan Bornschein, Shona MacRae, et al. 2016. “Mutational Signatures in Esophageal Adenocarcinoma Define Etiologically Distinct Subgroups with Therapeutic Relevance.” *Nat. Genet.* 48 (10): 1131–41.

  
  
  
**MEDICAL DISCLAIMER**:*The information contained in this cancer genome report is intended for research purposes only. We make no representations or warranties of any kind, expressed or implied, about the completeness, accuracy, reliability, suitability or availability with respect to the genome report or the information, products, services, for interpretation or use in clinical practice, or otherwise contained in the report for any purpose. Any reliance you place on information in the report is therefore strictly at your own risk. In no event will we be liable for any loss or damage including without limitation, indirect or consequential loss or damage, or any loss or damage whatsoever arising from loss of data or profits arising out of, or in connection with, the use of this genome report.*
