# Supplementary figures and images for: Personal Cancer Genome Reporter: variant interpretation report for precision oncology
Source: Bioinformatics. 2017 Dec 20;34(10):1778–80. doi: 10.1093/bioinformatics/btx817 (PMC5946881; doi:10.1093/bioinformatics/btx817)

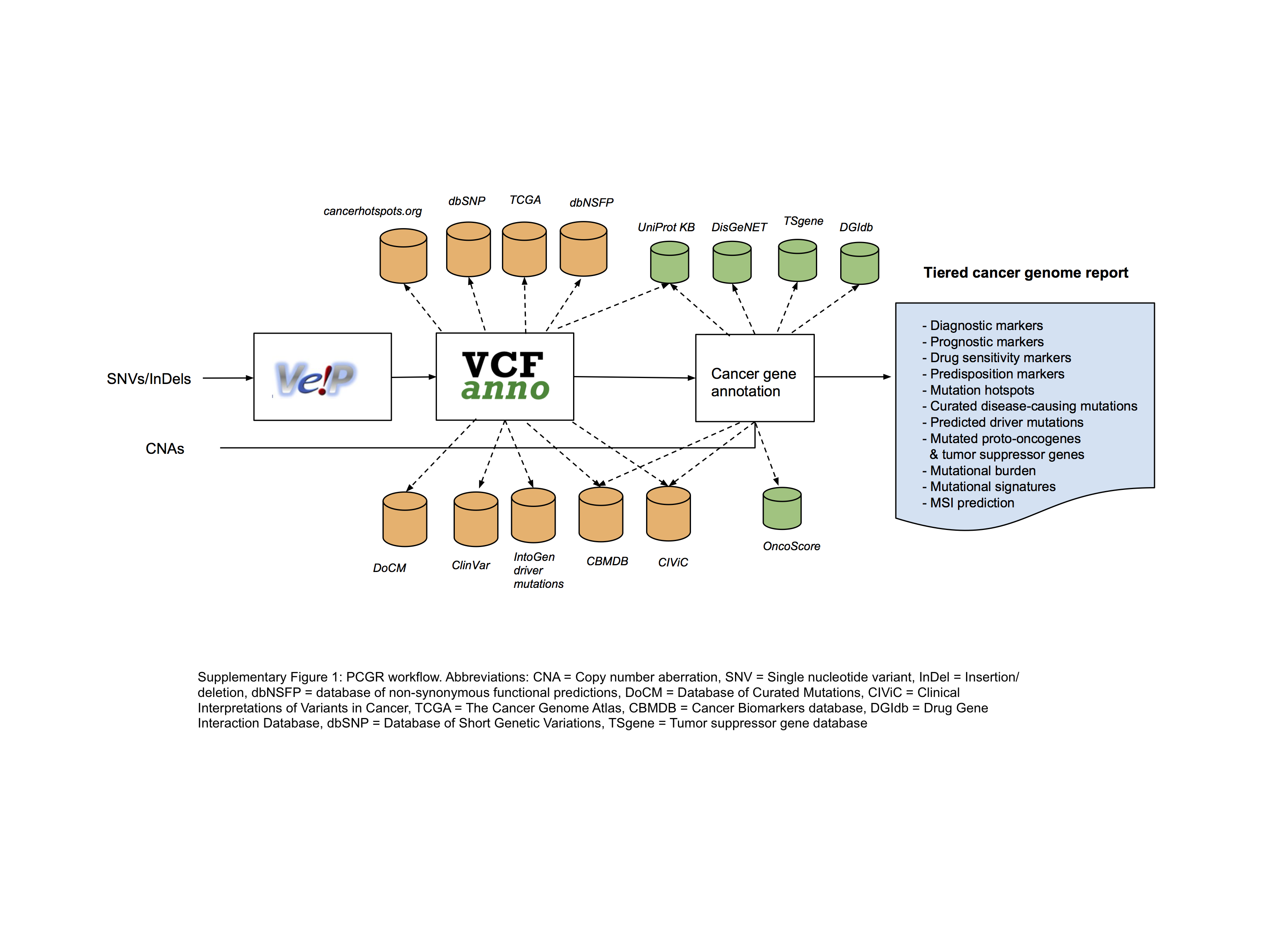

Supplement: Supplementary Data [file btx817_supp.zip › btx817-suppl_data/PCGR_workflow.v3.tiff]
